# Why is advance care planning underused in oncology settings? A systematic overview of reviews to identify the benefits, barriers, enablers, and interventions to improve uptake

**DOI:** 10.3389/fonc.2023.1040589

**Published:** 2023-04-28

**Authors:** Lisa Guccione, Sonia Fullerton, Karla Gough, Amelia Hyatt, Michelle Tew, Sanchia Aranda, Jill Francis

**Affiliations:** ^1^ Department of Health Services Research, Peter MacCallum Cancer Centre, Melbourne, VIC, Australia; ^2^ Sir Peter MacCallum Department of Oncology, Faculty of Medicine, Dentistry and Health Sciences, The University of Melbourne, Melbourne, VIC, Australia; ^3^ Department of Oncology, Parkville Integrated Palliative Care Service, Peter MacCallum Cancer Centre, Melbourne, VIC, Australia; ^4^ Department of Nursing, Faculty of Medicine, Dentistry and Health Sciences, The University of Melbourne, Melbourne, VIC, Australia; ^5^ Centre for Health Policy, Melbourne School of Population and Global Health, The University of Melbourne, Melbourne, VIC, Australia; ^6^ School of Health Sciences, The University of Melbourne, Melbourne, VIC, Australia; ^7^ Ottawa Hospital research Institute, Clinical Epidemiology Program, Ottawa, ON, Canada

**Keywords:** advance care planning (ACP), barriers and enablers, healthcare provider (HCP), improving uptake, patient-centered care, theoretical domains framework

## Abstract

**Background:**

Advance care planning (ACP) centres on supporting people to define and discuss their individual goals and preferences for future medical care, and to record and review these as appropriate. Despite recommendations from guidelines, rates of documentation for people with cancer are considerably low.

**Aim:**

To systematically clarify and consolidate the evidence base of ACP in cancer care by exploring how it is defined; identifying benefits, and known barriers and enablers across patient, clinical and healthcare services levels; as well as interventions that improve advance care planning and are their effectiveness.

**Methods:**

A systematic overview of reviews was conducted and was prospectively registered on PROSPERO. PubMed, Medline, PsycInfo, CINAHL, and EMBASE were searched for review related to ACP in cancer. Content analysis and narrative synthesis were used for data analysis. The Theoretical Domains Framework (TDF) was used to code barriers and enablers of ACP as well as the implied barriers targeted by each of the interventions.

**Results:**

Eighteen reviews met the inclusion criteria. Definitions were inconsistent across reviews that defined ACP (n=16). Proposed benefits identified in 15/18 reviews were rarely empirically supported. Interventions reported in seven reviews tended to target the patient, even though more barriers were associated with healthcare providers (n=40 versus n=60, respectively).

**Conclusion:**

To improve ACP uptake in oncology settings; the definition should include key categories that clarify the utility and benefits. Interventions need to target healthcare providers and empirically identified barriers to be most effective in improving uptake.

**Systematic review registration:**

https://www.crd.york.ac.uk/prospero/display_record.php?, identifier CRD42021288825.

## Introduction

A recent international consensus definition for advance care planning (ACP) states that ACP is “the ability to enable individuals to define goals and preferences for future medical treatment and care, to discuss these goals and preferences with family and health-care providers, and to record and review these preferences if appropriate” (1). Ultimately the goal of ACP is to align the treatment a person receives with their preferences for care (2). Despite practice guidelines recommending ACP for people with cancer, results from the Australia National Advance Care Directive Prevalence study (2017) suggested that only 27% of people with cancer had documented their ACP preferences in an advance care directive (3). This finding is consistent with low rates of ACP discussion and documentation reported internationally (4–6).

In Australia, the terms used in advance care planning differ by state. Nationally, the term ‘substitute decision maker’ is used to denote the person who makes medical decisions if a person loses medical decision-making capacity. ‘Advance care directive’ is the umbrella term for documents expressing the person’s preferences for future health care in the event that they lose medical decision-making capacity. Internationally there is considerable variation in terminology used for ACP. However, the principles of appointing a surrogate decision maker, having conversations about preferences and values, and recording a written advance care directive are generally applicable. In the USA, physician orders such as Do Not Attempt Resuscitation (DNAR) are included in ACP documentation (1). In Europe, concepts and laws regarding ACP differ, with some countries having legally binding frameworks and others not (1). Some examples from English-speaking countries and Europe are presented in **Supplementary File 1** (*Advance care planning terms of reference*). Often, laws regarding ACP are made at a state or provincial, rather than at a national, level. The lack of consistency in terms and definitions used can be confusing for patients and health providers.

Literature proposes a range of benefits of ACP across various populations. However, it is uncertain from the literature on cancer patients if proposed benefits of ACP have been empirically identified. Studies have found that the values and needs of cancer patients in response to ACP are different to other patient populations (7). For example, patients with cancer placed greater emphasis on decisions on their preferences for site or care rather than intervention-based treatment decisions (7). Also unknown from the literature is whether interventions to support uptake of ACP are targeting the most frequently reported barriers and enablers of ACP, and if so are they effective in improving uptake.

With several published reviews identifying barriers to ACP (8–10) and interventions to support uptake of ACP (11, 12), the aim of this overview of reviews is to clarify and consolidate the evidence base in oncology settings to inform recommendations for improving uptake of ACP. This overview took a systematic approach to searching, appraising, and synthesizing the review literature to address the following research questions (13):

1. How has advance care planning (ACP) been defined and what are the included elements?
2. What are the proposed and empirically supported benefits of ACP in oncology settings?
3. What are the known barriers and enablers of ACP uptake across patient, clinician, healthcare service, and systems levels?
4. Which interventions to improve ACP uptake have been reported, do they target the identified barriers and enablers, and how effective are they?

## Methods

This systematic overview of reviews used a standardized protocol prepared according to the Preferred Reporting Items for Systematic reviews and Meta-Analyses (PRISMA) guidelines (14). The protocol was registered with Prospero; registration number: CRD42021288825 (https://www.crd.york.ac.uk/prospero/display_record.php?ID=CRD42021288825).

### Search strategy

The search was conducted by one author (LG) using databases PubMed, Medline, PsycInfo, CINAHL, and EMBASE. Papers were restricted to reviews published in English, within a 10-year publication date range from 2011 to August 4, 2021. The search strategy was designed in collaboration with an expert librarian and critically discussed by the research team, capturing terms and synonyms relating to three domains: “advance care plan”, “cancer” and “review”. A full list of search terms is provided in **Supplementary File 2**.

### Inclusion and exclusion criteria

A review was included in this systematic overview of reviews if it fulfilled all the following inclusion criteria: (1) published in a peer-reviewed journal; (2) English language; (3) reported only on the populations of interest: adult cancer patients of any gender, healthcare providers responsible for facilitating ACP with adult cancer patients, or family or caregivers of adult cancer patients; and (4) reported on ACP using any definition from the perspectives of patients, healthcare professionals, or staff at hospital service or system levels. We excluded an article if it: (1) reported on a pediatric cancer population; (2) focused on community settings; or (3) did not address at least one of the research questions.

### Screening and selection of the literature

All identified reviews were uploaded to EndNote (15) and imported into Covidence (16) to manage citations and remove duplicates. Following de-duplication, two authors (LG and SF) screened identified articles to determine eligibility for inclusion. Screening occurred in two steps: an initial screen of titles and abstracts against the eligibility criteria, and a further step of retrieving the full paper if eligibility could not be confirmed from the abstract. Screening involved judging each review as either: eligible, not eligible, or potentially eligible. Conflicts were resolved initially through discussion (LG and SF) and presented to the research team for final resolution. All differences of opinion were resolved by consensus.

### Data extraction and analysis

Data extraction templates were designed to enable extraction of all data addressing the research questions and to facilitate consistency of extraction across studies and reviewers. For all reviews that met the inclusion criteria, data extraction was conducted by one author (LG), with 20% of the reviews crosschecked by a second author (SF).

Content analysis (17, 18) and narrative synthesis (19) were used to organise and summarise ACP definitions (research question 1), and the proposed and empirically supported benefits of ACP (research question 2). Proposed benefits were those that formed part of the rationale of an included review, and empirically identified benefits, were those that reported measured outcomes of ACP. These analyses were conducted by one author (LG) and reviewed by a second author (AH or KG).

Reported barriers and enablers of requesting and recording ACP details from a healthcare professional perspective, or deciding and communicating ACP details from a patient perspective were coded into the Theoretical Domains Framework (TDF) (20) (research question 3). This framework was developed to synthesise 33 theories of behaviour, to provide a theory-informed basis for identifying barriers and enablers of behaviour (21, 22). The thematic analysis was conducted by one author (LG) and reviewed by a second author (JF). Identified themes were assessed against previously published ‘importance criteria’ to determine the likely importance and role of each domain in influencing behaviours related to ACP (23). These criteria were: frequency (number of reviews that identified each domain; elaboration (number of content themes identified in each domain); and ‘expressed importance’ (statement from the authors expressing importance in relation to ACP uptake).

Content analysis (17, 18) was used to organize and summarise the details of the interventions such as the various forms of delivery and intervention content. The implied barriers targeted by each of these interventions were coded to the Theoretical Domains Framework domains. For example, educational interventions imply that lack of knowledge is a barrier, whereas communication skills training implies that lack of skills or lack of confidence to discuss ACP are barriers, even if these assumptions are not explicit. This analysis was conducted by one author (LG) and reviewed by another author (JF). We also report on evidence of effectiveness of these interventions for improving documentation of ACP (research question 4).

### Quality assessment

The Joanna Briggs Institute (JBI) critical appraisal assessment checklist for systematic reviews was used to assess the methodological quality of the systematic and scoping reviews included in this overview of reviews. This checklist consists of 10 items that address methodological characteristics of a review article including: appropriateness of search strategies, potential sources of bias, and prospects for future research and policy-making (24). Each item is scored as 1 (*met)* or 0 (*not met, unclear, or not applicable*) with item scores summed to calculate an overall score. Studies scoring 0-4, 5-7 and 8-10 points were categorized as low, medium, and high quality, respectively, as described by Hossain et al. (25).

The methodological quality of narrative reviews included in this overview was assessed using the Scale for the Quality Assessment of Narrative Review Articles (SANRA) tool. The SANRA is a 6-item scale whereby each item is scored as 0 (*low quality*) to 2 (*high quality*), with item scores then summed; hence, the range of overall quality scores is 0-12. (Reviews scoring ≥9 are classified as high quality) (26).

All quality assessments were conducted by one reviewer (LG), with a second reviewer (SF) independently assessing a random selection of 20%. Minor differences in assessment were identified and discussed to reach consensus, or discussed with a third author (KG or MT). Reviews were not excluded based on quality assessment scores but any findings from reviews that received low scores were noted.

## Results

### Search results

**Figure 1:**
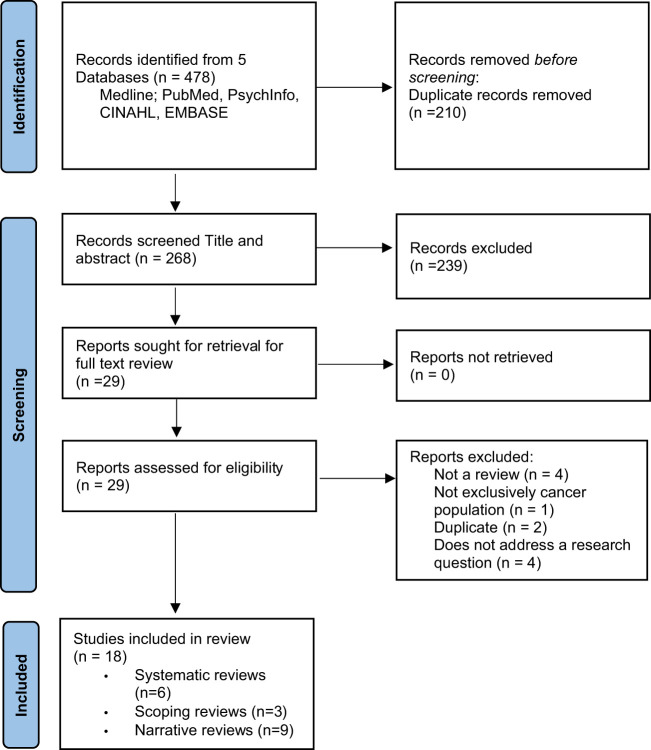
Preferred Reporting Items for Systematic Reviews and Meta-Analyses (PRISMA) flow diagram.

A total of 478 records from MEDLINE (n=56), PubMed (n=208), PsycInfo (n=27), CINAHL (n=92) EMBASE (n=95) were identified across all searches. Of these, 210 duplicates were removed. Following review of titles and abstracts, 29 records met the eligibility criteria and were retained for full text review. A further 11 records were excluded at initial full-text review, resulting in 18 records (12, 27–43) being included in the analysis (**Figure 1**).

### Characteristics of included reviews and quality appraisal

The included reviews consisted of systematic reviews (n=6), scoping reviews (n=3) and narrative reviews (n=9), published from 2011 to August 4, 2021, with more than half of these in the period 2018-2020. The majority used mixed-methods with only four using purely quantitative methods. The reviews mostly included both patients and healthcare providers (n=10), with seven reviews involving patients only and one review of healthcare providers only.

All systematic reviews included in this overview were appraised as high quality. The three scoping reviews, also assessed using the JBI checklist, were appraised as low (n=1), medium (n=1), and high quality (n=1). The main criteria leading to low scores included unclear search strategy, poorly defined or missing inclusion criteria, and no appraisal of included studies, which is likely due to a lack of standardized reporting for scoping reviews whereby these details are often omitted (44).

SANRA scores for narrative reviews ranged from 5-10 points, with a median score of 9 points. Although there are no predefined quality categories for this scale, experience suggests a score ≤4 is indicative of poor quality (26). Study characteristics and quality assessment scores are summarized in **Table 1**.

**Table 1:** Characteristics of included reviews and advance care planning (ACP) definition.

Study	Type of Review	Method of synthesis/ analysis	No., and timeframe of databases searched	No., types of publication., timeframe of primary studies	Country of the primary studies	Sample Characteristics. (patients and/ healthcare providers; terminal/ non-terminal, settings)	Quality assessment * JBI critical appraisal; SANRA appraisal	Definition of ACP
Argarwal et al. (38)	Narrative Review	Narrative	Not Reported	Not reported; quantitative and qualitative; 1996-2017	Not reported	Patients and healthcare providers; unclear if terminal or non-terminal; inpatient and outpatient settings	SANRA Score 10	“*the multifaceted process by which patients make decisions regarding their future medical care. Oncologists and palliative care specialists have shared responsibility to candidly discuss prognosis and recommend end-of-life care options at appropriate times in a cancer patient’s disease trajectory. The primary objective … to enable patients to cogitate about their goals at the end of life, and in turn, make informed health care related choices that are congruent with and fulfil these wishes.*”
Argarwal et al. (43)	Narrative Review	Narrative	1 database; Not reported	Not reported; quantitative and qualitative; 1991-2017	Not reported	Patients and healthcare providers; unclear if terminal or non-terminal; patient settings unclear	SANRA Score 9	*“an essential process by which patients with serious illnesses are empowered to articulate their personal values, preferences, and goals to make decisions for their future care… having conversations between patients, providers, and families, it should ultimately lead to documentation of patients’ wishes, beliefs, and values by way of completing an advance directive while the patient still has decisional capacity.”*
Bestvina et al. (39)	Narrative Review	Narrative	1 database; 2006-2013	26; quantitative; 2007-2017	Australia, USA, Europe,	Patients and healthcare providers; non terminal; outpatient settings	SANRA Score 8	*“process whereby a patient, in consultation with health care providers, family members, and important others, makes decisions about his or her future health care.”*
Buiar et al. (32)	Scoping Review	Narrative	1 database; 1979-2019	Not reported; quantitative and qualitative; 1987-2018	Not reported	Patient and healthcare providers; unclear if terminal or non-terminal; both inpatient and outpatient settings	Low-quality (JBI)	None provided.
Cohen et al. (35)	Narrative Review	Narrative	Not reported	Not reported; quantitative and qualitative; 2002-2011	USA	Patients and healthcare providers; unclear if terminal or non-terminal; inpatient settings	SANRA Score 10	*“a process that allows people to make decisions in advance regarding their medical treatment at the end of life (EOL)… includes clear discussions about prognosis, information about ADs, explanation of the do-not-resuscitate (DNR) option, information about palliative care options (e.g., hospice), and discussion about where patients would prefer to die. ADs are legal documents representing those decisions and can be statements written by patients about how they want their medical decisions made (i.e., living will) or whom they would like to make decisions if they are no longer able to make them themselves (i.e., power of attorney or healthcare proxy), or a combination of both (i.e., Five Wishes document).”*
Dirven et al. (37)	Narrative Review	Narrative	Not Reported	Not reported; quantitative and qualitative; 1996-2014	Not reported	Patients only; terminal; inpatient settings	SANRA Score 7	*“a process in which patients, in consultation with their families and physicians, make decisions regarding future goals of the EOL care. These discussions may result in the completion of an advance directive.”*
Johnson et al. (27)	Systematic Review	Thematic	5 databases; from inception to November 2014	40; quantitative and qualitative; 1996-2014	USA, UK, Europe, Australia, Taiwan and Canada	Patients and healthcare providers; unclear if terminal; both inpatient and outpatient settings	High-quality (JBI)	*“formalised discussion between patients and healthcare providers which may include family members or friends, with possible outcome of formal documentation of EOL care wishes.”*
Karlin et al. (40)	Narrative Review	Narrative	1 database; Not reported	Not reported; quantitative and qualitative; 1991-2017	Not reported	Patients only; terminal; patient settings unclear	SANRA Score 5	None provided
Khan et al. (36)	Narrative Review	Narrative	1 database; Not Reported	Not reported; quantitative and qualitative; 1990-2013	Not reported	Patients only; terminal; patient settings unclear	SANRA Score 9	*“Discussing and documenting patients’ preferences for their end-of life care through advance care planning is a key component of palliative care.”*
Kuusisto et al., (33)	Scoping Review	Content Analysis	4 databases; None applied	12;quantitative and qualitative; 2010-2019	USA, Europe, Asia and Australia	Healthcare providers only; non terminal; both inpatient and outpatient settings	High-quality (JBI)	*“a multifaceted, family-centred and social process by which patients make decisions regarding their future medical care. The primary goal ….. is to enable patients to consider their goals at the end of life so that they will receive the care they desire … their preferences can be taken into account even if they are unable to make their own decisions. The starting point… should be patients’ right to self-determination … Documentation of … conversations and/or completion of legal documents … is recommended … refers to both oral discussion (advance care planning) and written document (advance care plan).”*
Levoy et al. (12)	Systematic Review	Realist approach	5 databases; 1990-2018	25; quantitative; 2007-2018	USA, Australia, UK, Switzerland, China,	Patients only; non terminal; both inpatient and outpatient settings	High-quality (JBI)	*“an essential aspect of providing patient-centred care to those with an advanced serious illness, such as cancer….has three main components: completing a living will, designating a health care surrogate, and participating in end-of-life (EOL) discussions….is not a one-time event, but rather a process that evolves over the patient’s illness trajectory to match care to the patient’s goals and values….should be initiated early in the illness trajectory and routinely reviewed when changes in the patient’s condition or transitions of care occur.”*
Lin et al. (41)	Narrative Review	Narrative	Not Reported	Not reported; quantitative and qualitative; 1991-2018	Not reported	Patients and healthcare providers; non terminal; in-patient and outpatient settings	SANRA Score 10	*“a voluntary process that supports adults at any age or stage of health who possess mental capacity (the ability to make a decision for him- or herself) in understanding and sharing their personal values, life goals, and preferences regarding future (medical) care. It is an ongoing process of assessment and communication among patients, family members, healthcare professionals and medical surrogates to reach a consensus on medical care for patients, and it consists of written documents such as advance directives/decisions (ADs) or advance statement (AS)… usually used in the context of progressive illness and anticipated deterioration, and it greatly varies from general care planning.”*
Lin et al. (29)	Systematic Review	Narrative	8 databases; from inception to March 2017	9; quantitative; 2007-2017	USA, UK, Australia	Patients only; terminal; both inpatient and outpatient settings	High-quality (JBI)	*“ensuring patients’ access to preferred care, by conducting a mutual communication between patients, families and healthcare professionals to achieve consensus on future care.”*
Marchi et al., (31)	Systematic Review	Thematic	4 databases; from inception to March 2018	7; quantitative and qualitative; 2011-2018	Not reported	Patient and healthcare providers; terminal; settings unclear	High-quality (JBI)	*“a decision-making process for future health care for patients undergoing treatment that includes the effective participation of physicians, family members, and other people considered important in this treatment. It aims to ensure that patients’ desires are respected when they are no longer able to make decisions…. provides the possibility for patients to be involved in and decide about treatments that he or she wants or does not want at the end of life, in addition to electing a family member or people closer to him or her who can make decisions in a shared manner, ultimately recording their decisions by means of advance directives (ADs) of will or through Physician Orders for Life-Sustaining Treatment (POLST)”*
Matsuoka et al. (42)	Narrative Review	Narrative	Not Reported	Not reported; quantitative and qualitative; 1994-2018	Not reported	Patients and healthcare providers; unclear if terminal or non-terminal; patient settings unclear	SANRA Score 6	*“the process whereby patients consult with health care professionals, family members and other loved ones to make individual decisions about their future healthcare and medical treatments to prepare for when patients lose competency to express their wishes… enables patients and their families to consider what care and treatments might or might not be acceptable, and to implement care and treatment consistent with their wishes…. primarily focuses on planning for the time when patients are incapable of making a decision, but it can also be applied to patients who retain capacity. Originally… was implemented to complete written documents, such as advance directives (ADs), do-not-resuscitate (DNR) orders and do-not-hospitalize (DNH) orders. Nowadays, the focus… is regarded as not only about the completion of written forms but also on the social process of communication between patients and care providers.”*
Song et al. (28)	Systematic Review	Narrative	8 databases; from inception to July 2016	19; quantitative and qualitative; 2000-2016	USA, Italy, Australia, Germany, Austria, Netherlands, Austria and the UK	Patients only; unclear if terminal or non-terminal; both inpatient and outpatient settings	High-quality (JBI)	*“the ongoing process that involves decisions made by patients, in consultation with surrogate decision-makers, family and health care providers regarding their values, beliefs, life-sustaining treatment preferences, goals of care (GOC), and palliative care options, should they later become incapable of expressing such wishes….may further include the patient completing an advance directive (AD) which documents his or her wishes and/or appointment of a substitute decision-maker.”*
Spelton et al. (34)	Scoping Review	Thematic	4 databases; 2013-2018	11; quantitative and qualitative; 2013-2018	Mostly USA	Patients only; unclear if terminal or non-terminal; both inpatient and outpatient settings	Medium-quality (JBI)	*“a patient's decisions about prospective health care, in consultation with family members and healthcare providers. The aim is to empower patients in anticipation of a decline in their health, ready to be referred to if they become unable to convey their wishes or make decisions about their medical treatment.”*
Starr et al. (30)	Systematic Review	Narrative	3 databases; January 2012-January 2019	20; quantitative; 2012-2019	USA	Patient and healthcare providers; terminal; both inpatient and outpatient settings	High-quality (JBI)	*“discussions about patient values, prognosis, treatment options, aspects of living and dying, or specific interventions a patient may want if certain future conditions occur… conversation about EOL goals or treatment preferences with a healthcare provider or trained facilitator, documented in medical records or self-reported by patients or surrogates… sometimes includes advance directives (AD), physician orders for life-sustaining treatment (POLST), or do-not-resuscitate (DNR) or do-not-intubate (DNI) orders that suggest discussion about preferences”*

*JBI: Joanna Briggs Institute, quality assessment of systematic reviews (19); SANRA: Scale for the Assessment of Narrative Review Articles (21).

### How has ACP been defined?

ACP was defined in 16 of the 18 reviews (12, 27–31, 33–39, 41–43). The systematic (32) and narrative review (40)without a definition of ACP both scored the lowest on the JBI and SANRA quality assessment tools, respectively.

**Figure 2:**
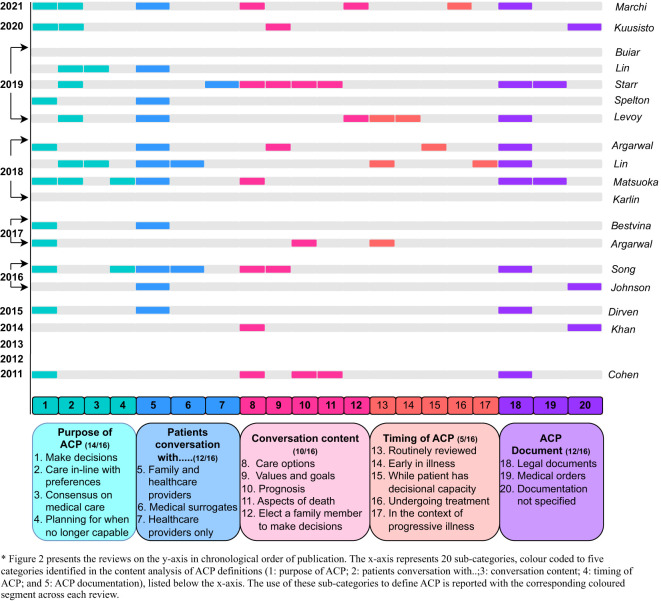
Chronological mapping of categories identified in the content analysis of ACP definitions across reviews.

**Figure 2** presents the content categories and sub-categories used to define ACP across all reviews, listed chronologically. Overall, it appears that a consistent definition of ACP has not developed over time. The most common combination of categories and subcategories used in defining ACP were as follows: ‘the purpose of ACP is—to make decisions’; ‘patients should have conversations with—family and healthcare providers’; conversations should cover—care options’; and ‘ACP should result in documentation—in the form of a legal document’. Notably, prior to 2017, the timing of ACP development was not included in any definition, and once present, not consistently described; although in the context of oncology settings, reviews included both terminal and non-terminal cancer patients (terminal patients only, n=6; non-terminal patients, n=4; and unclear, n=8).

## Proposed and identified benefits of ACP

Content analysis identified three categories of proposed and empirically supported benefits of ACP: patient benefits, family benefits, and healthcare service benefits, presented in **Table 2**.

**Table 2:** Summary of proposed and identified benefits of ACP by content categories and frequencies of sub-categories for each.

Proposed benefits (14/18)	Empirically supported benefits (8/18)	Proposed benefits that were then empirically supported in the same review (6/18)
Patient Benefits(13/14): Care aligned with patient preferences (12, 28, 29, 31, 34, 35, 38, 39, 41, 43) Satisfaction (12, 29, 28, 33, 37) Patient empowerment (27, 38, 41, 43) Quality of life (29, 34, 39) Quality care (3/14), and 34, 35, 41) Patient information (27, 35)	Patient Benefits *(5/8)* Quality care (28, 31, 41) Care aligned with patient preferences (28, 31) Patient information (27) Quality of life (41) ACP not associated with depression (40)	Patient Benefits (4/6) Care aligned with patient preferences (28, 31) Patient Information (27) Quality care (28, 41)
Family Benefits (8/14): Psychological benefits (12, 28, 31, 33, 39) Satisfaction with care (28, 29, 31) Caregiver quality of life (35) Impact on bereavement (34)	Family Benefits (2/8): Psychological benefits (41) Satisfaction with care (28)	Family Benefits (1/6) Satisfaction with care (28)
Healthcare Service Benefits (11/14): Reduced hospitalisations (12, 28, 29, 33, 38, 43) Increased hospice (12, 28, 35, 38, 39, 43) Reduced cost of care (29, 34, 30, 37, 39) Reduced aggressive care/life sustaining intervention (12, 30, 35, 39) Reduced treatment (33, 38, 43 Decreased hospital deaths (33, 43)	Healthcare Service Benefits (7/8): Reduced hospitalisations (28, 30, 31, 36, 41) Increased hospice (30, 31, 40, 41) Reduced aggressive care/life sustaining intervention (39–41) Reduced cost of care (40, 41, 30) Reduced treatment (*Marchi, 2020)*	Healthcare Service Benefits (3/6) Reduced hospitalisations (28) Reduced aggressive care/life sustaining intervention (39) Reduced cost of care (31)

A misalignment was found between the proposed and empirically supported benefits of ACP, with many proposed benefits for patients, families, and healthcare providers not empirically supported within the same review. In terms of patient benefits, only ‘quality care’, ‘patient information’ and ‘care alignment’ had both proposed and empirically supported benefits (27, 28, 31, 41). For families, only ‘satisfaction with care’ was proposed as a benefit and empirically supported in the same review (28). Assessment of health care service benefits identified reduced hospitalization, reduced aggressive care, and reduced cost of care, as both proposed and empirically supported (28, 30, 39).

## Barriers and enablers of requesting, recording, deciding, and or communicating ACP

A deductive analysis identified barriers and enablers of ACP across 12 of the 14 Theoretical Domains Framework domains from 15/18 reviews (12, 27–29, 32–35, 37–43). **Table 3** presents frequencies of the barriers and enablers by Theoretical Domains Framework domain, and content themes identified within each domain from the patient, healthcare provider, and healthcare service perspectives. More barriers of ACP were associated with healthcare providers (n=60) in comparison to patients (n=40) and healthcare services (n=3). Enablers of ACP were more frequently identified for patients (n=17) compared to healthcare providers (n=15) and healthcare services (n=3).

**Table 3:** Theoretical Domains Framework domains and themes of barriers and enablers across patient, healthcare provider, and healthcare services perspectives.

Theoretical Domains Framework domains and themes	Review References	Frequencies		Patient Perspective		Healthcare Provider Perspective		Healthcare Services Perspective	
		Barriers	Enablers	Barriers	Enablers	Barriers	Enablers	Barriers	Enablers
*Knowledge*		*17*	*7*	9	3	8	4	0	0
• Procedural understanding or lack of knowing when, how, or what to discuss	(30, 35, 38)	2	1	**-**	**-**	**X**	**X**	**-**	**-**
• Clinician certainty of prognosis or lack of	(35, 40)	2	1	**-**	**-**	**X**	**X**	**-**	**-**
• Patient understanding, or lack of understanding of their prognosis	(26, 29, 31, 35, 40)	3	2	**X**	**X**	**X**	**X**	**-**	**-**
• Clinician ignorance as a knowledge deficit	(24)	1	0			**X**		**-**	**-**
• Clinicians understanding of patient preferences of care	(39)	0	1	**-**	**-**	**-**	**X**	**-**	**-**
• Patient lack of knowledge of their disease	(29, 39)	2	0	**X**	**-**	**-**	**-**	**-**	**-**
• Medical knowledge or lack of	(29, 35, 25)	1	1	**X**	**X**	**-**	**-**	**-**	**-**
• Understanding of ACP and its use or non-use	(26, 24, 29–32)	6	1	**X**	**X**	**X**	**-**	**-**	**-**
*Environmental context and resources*		*10*	*6*	2	2	5	1	3	3
• Time constraints associated with outpatient and inpatient settings	(24, 35, 38, 40)	5	1	**X**	**X**	**X**	**-**	**-**	**-**
• System level constraints in accessing information	(26, 29, 30, 32, 39)	3	3	**-**	**-**	**-**	**-**	**X**	**X**
• Privacy – appropriateness of settings to have the conversation	(24, 26, 31)	2	2	**X**	**X**	**X**	**X**	**-**	**-**
*Emotion*		*17*	*1*	8	1	9	0	0	0
• Fear of impacting the therapeutic relationship	(35)	1	0	**-**	**-**	**X**	**-**	**-**	**-**
• Diminishing hope	(24, 32, 35, 37–39)	5	0	**-**	**-**	**X**	**-**	**-**	**-**
• Fear of addressing bad news	(29)	1	0	**-**	**-**	**X**	**-**	**-**	**-**
• Patient stress and anxiety	(24, 29, 31)	3	0	**X**	**-**	**X**	**-**	**-**	**-**
• Patient fear	(24)	1	0	**X**	**-**	**-**	**-**	**-**	**-**
• Patient fear for family members	(24)	1	0	**X**	**-**	**-**	**-**	**-**	**-**
• Perceived physician discomfort in initiating discussions	(24, 32)	3	0	**X**	**-**	**-**	**-**	**-**	**-**
• Acceptance of prognosis and realistic expectations or lack of	(32, 40)	2	1	**X**	**X**	**X**	**-**	**-**	**-**
*Skills*		*8*	*5*	1	2	7	2	0	0
• Training on ACP, or lack thereof (2)	(32, 35–36, 39–40)	5	1	**-**	**-**	**X**	**X**	**-**	**-**
• Lack of training in navigating systems to retrieve ACP information	(30)	1	0	**-**	**-**	**X**	**-**	**-**	**-**
• Palliative care skills to aid discussions of ACP preparation/readiness	(40)	0	1	**-**	**-**	**-**	**X**	**-**	**-**
• Clinician communication skills (2)	(24–26, 35, 39)	2	3	**X**	**X**	**X**	**X**	**-**	**-**
*Social/professional role and identity*		*13*	*3*	7	0	6	3	0	0
• Role clarity or lack thereof	(24, 30, 36)	3	0	**-**	**-**	**X**	**-**	**-**	**-**
• Nurses lack of perceived authority in decisions with EoL care	(24)	1	0	**-**	**-**	**X**	**-**	**-**	**-**
• Nurses perception that others (patients/family/doctors) didn’t think it was their role	(32)	1	0	**-**	**-**	**X**	**-**	**-**	**-**
• Perception of patient/physician relationship	(24)	1	0	**-**	**-**	**X**	**-**	**-**	**-**
• Multidisciplinary approach	(10, 30, 32)	0	3	**-**	**-**	**-**	**X**	**-**	**-**
• Patients feeling it is not their role to make decisions	(24)	1	0	**X**	**-**	**-**	**-**	**-**	**-**
• Cultural and/or religious beliefs	(31, 34, 38)	6	0	**X**	**-**	**-**	**-**	**-**	**-**
*Beliefs about consequences*		*19*	*3*	5	2	14	1	0	0
• Having the conversation at the wrong time/patient readiness	(29, 38, 40)	2	1	**-**	**X**	**X**	**-**	**-**	**-**
• Discussion would have a negative impact	(24, 29, 37–40)	6	0	**-**	**-**	**X**	**-**	**-**	**-**
• Conversation will damage the patient/physician relationship	(24)	1	0	**-**	**-**	**X**	**-**	**-**	**-**
• Nurses beliefs on repercussions from doctors for initiating ACP conversation	(24, 32)	2	0	**-**	**-**	**X**	**-**	**-**	**-**
• Nurses feeling that patients/families do not want to have the conversation with them	(32)	1	0	**-**	**-**	**X**	**-**	**-**	**-**
• Patient perception that ACP will impact receiving adequate care	(32)	1	0	**X**	**-**	**-**	**-**	**-**	**-**
• Patient unsure if ACP is useful	(32)	1	0	**X**	**-**	**-**	**-**	**-**	**-**
• Patient perception that ACP conversation will upset family members	(24)	1	0	**X**	**-**	**-**	**-**	**-**	**-**
• Past experiences and attitudes towards the health care system	(24, 38)	4	2	**X**	**X**	**X**	**X**	**-**	**-**
*Social influences*		*9*	*10*	6	6	3	4	0	0
• Clinician discussions with other colleagues that share responsibilities of the patient	(24)	0	1	**-**	**-**	**-**	**X**	**-**	**-**
• Consideration of culturally appropriate was to engage in in ACP conversation	(38)	0	1	**-**	**-**	**-**	**X**	**-**	**-**
• Exclusion from ACP conversations	(29, 31, 35)	3	0	**X**	**-**	**-**	**-**	**-**	**-**
• Family participation in ACP conversation	(24, 39)	1	2	**X**	**X**	**-**	**X**	**-**	**-**
• Institutional culture	(24, 38)	4	0	**X**	**-**	**X**	**-**	**-**	**-**
• Clinician engagement in ACP conversation	(24, 35, 38– 39)	2	6	**-**	**X**	**X**	**X**	**-**	**-**
*Behavioural regulation*		*4*	*0*	0	0	3	0	0	0
• No guideline established for the timing of ACP	(30, 35–36)	3	0	**-**	**-**	**X**	**-**	**-**	**-**
*Memory, attention and decision processes*		*4*	*1*	2	1	2	0	0	0
• Not disclosing poor prognosis to patients	(29, 31, 38)	2	0	**-**	**-**	**X**	**-**	**-**	**-**
• Knowing when is the best time to initiate ACP conversations	(29–30)	1	1	**-**	**X**	**X**	**-**	**-**	**-**
• Patients capability to make decisions	(29)	1	0	**X**	**-**	**-**	**-**	**-**	**-**
*Intentions*		*2*	*0*	0	0	2	0	0	0
• Reluctance towards early initiation of ACP	(30, 37)	2	0	**-**	**-**	**X**	**-**	**-**	**-**
*Goals*		*2*	*0*	0	0	2	0	0	0
• Waiting for ACP to be relevant	(24)	2	0	**X**	**-**	**X**	**-**	**-**	**-**
*Optimism*		*1*	*0*	0	0	1	0	0	0
• Not wanting to discuss EoL unless patient is near death	(32)	1	0	**-**	**-**	**X**	**-**	**-**	**-**
*Behavioural capabilities*	*-*	*0*	*0*	-	-	-	-	-	-
*Reinforcement*	*-*	*0*	*0*	-	-	-	-	-	-

X denotes domain/themes identified.

As described in the method, we assessed importance in relation to three previously published criteria: domain frequency, level of elaboration within each domain, and authors’ explicit statements about importance (22, 23). Of 14 possible domains, the most frequently coded across the 15 reviews were: knowledge (66%); environmental context and resources (66%); emotion (66%); skills (60%); social/professional role and identity (53%); beliefs about consequences (46%); and social influences (40%). High levels of elaboration were found in the most frequently coded domains, except for those where minimal themes are to be expected: for example, skills whereby communication and training were predominant.

Evidence of importance was further supported by the authors of reviews articulating specific barriers or enablers as important in influencing ACP; for example, *“Health professionals’ knowledge of and attitudes towards ACP was also consistently found to be an important factor in their willingness to initiate or participate in ACP”* (27). Importance was also inferred in statements that articulated the patient’s voice; for example, *“Patients generally preferred to do ACP with the physician who knows them best, preferred that their physicians initiated discussion regarding ACP, and were more likely to participate in ACP or draw up an advance directive (AD) if they had discussed this with their oncologist” (* 27).

Statements of expressed importance were identified in seven reviews (27, 29, 32, 35, 39, 42, 43) and these aligned with the seven most frequently coded domains with the greatest level of elaboration: knowledge (n=5); skills (n=4); environmental context and resources (n=4); social influences (n=3); beliefs about consequences (n=2); social/professional role and identity (n=2); and emotion (n=1). High frequency content themes within these domains also aligned with expressed statements of importance. Details on domains of importance and example quotations are presented in **Table 4**. **Supplementary File 3** presents a narrative description and sample quotations for all themes across Theoretical Domains Framework domains.

**Table 4:** Theoretical Domains Framework domains of importance in influencing behaviours related to ACP; identified themes and quotations of expressed importance.

Theoretical Domains Framework Domain and level of elaboration	Themes of importance	% frequency of coded theme for domain	Examples of quotations of expressed importance or patient voice	Reviews with statements of expressed importance
**Knowledge** **(8 themes)**	*Understanding of ACP and its use or non-use*	29%	*“highlighted the importance of actively educating patients prior to the regular oncology consultation to enhance their ‘motivation’ and ‘competence’ to take part in an ACP discussion rather than just providing information on ACP to them”* (29)	(27, 29, 32, 42, 43)
			*Health professionals’ knowledge of … ACP was also consistently found to be an important factor in their willingness to initiate or participate in ACP”* (27)	
	*Patient understanding, or lack of understanding of their prognosis*	21%	“*Meaningful ACP requires good illness understanding and realistic expectations about prognosis so that patients can express their values and make decisions in a timely manner”* (43)	
			“*main reason patients fail to complete their directives is the difficulty in anticipating their wills based on scenario projections”* (32)	
**Environmental context and resources** **(3 themes)**	*System level constraints*	*37%*	*“most importantly a supportive contextual environment (e.g. availability of administrative system, sufficient resources… should be in place to support the implementation”* (29)	(27, 29; 32; 42)
	*Time constraints*	37%	**Patient expressed importance** *“ACP conversations should be initiated with adequate time and place for reflection”* (32)	
	*Appropriateness of setting*	*26%*	*“physicians reported time and privacy as barriers to ACP, they did so because they believed these were fundamental to establishing relationships with patients and families”* (27)	
**Emotion** **(8 themes)**	*Diminishing hope* *Acceptance of prognosis and realistic expectations*	28% 17%	*“Although nurses are well positioned to assist patients in ACP, barriers exist that prevent nurses from supporting patients in this way. The top reasons nurses did not discuss prognosis and hospice referral with their patients were unwillingness of patients or families to accept prognosis…..and nurses’ desire to maintain hope for patients and their families”* (35)	(35)
**Skills** **(4 themes)**	*Training on ACP or lack thereof*	46%	*“training in conducting ACP conversations should be offered to health care providers, as providers report feeling inappropriately trained or prepared to have ACP conversations”* (39)	(27, 29, 39, 42)
	*Clinician communication skills*	38%	*“communication and coaching skills training for medical staff were identified as essential requirements for successful ACP implementation”* (29	
**Social /professional role and identity** **(7 themes)**	*Role clarity or lack thereof*	*19%*	**Patient expressed importance** *- “patients generally preferred to do ACP with the physician who knows them best, preferred that their physicians initiated discussion regarding ACP, and were more likely to participate in ACP”* (27)	(27, 35)
**Beliefs about consequences** **(9 themes)**	*Discussion would have a negative impact*	30%	**Patients expressed importance contradicts this –** *“important to bear in mind that the majority of patients do not complain about additional depression/anxiety”* (32)	(32; 27)
	*Past experiences and attitudes towards the healthcare system*	27%	*“Health professionals’ knowledge of and attitudes towards ACP was also consistently found to be an important factor in their willingness to initiate or participate in ACP”* (27)	
**Social Influences** **(6 themes)**	*Clinician engagement in ACP conversation*	42%	**Patient expressed importance** *“preferred that their physicians initiated discussion regarding ACP, and were more likely to participate in ACP or draw up an AD if they had discussed this with their oncologist”* (27)	(32; 27, 42)
	*Institutional culture*	*21%*	*“The behaviour and choices of patients, their loved ones, and the staff caring for them in relation to EOL are strongly influenced by the institutional culture within which they are operating”* (27)	
	*Exclusion from ACP conversations*	*16%*	*“it is very important to reinforce that the directives should be ideally created by the patient themselves”* (32)	
	*Family participation in ACP conversations*	16%	*“Five essential elements of ACP for success (HP)* *Involvement of family in discussions…”* (42)	

## ACP Interventions – the barriers they address and effectiveness

**Figure 3:**
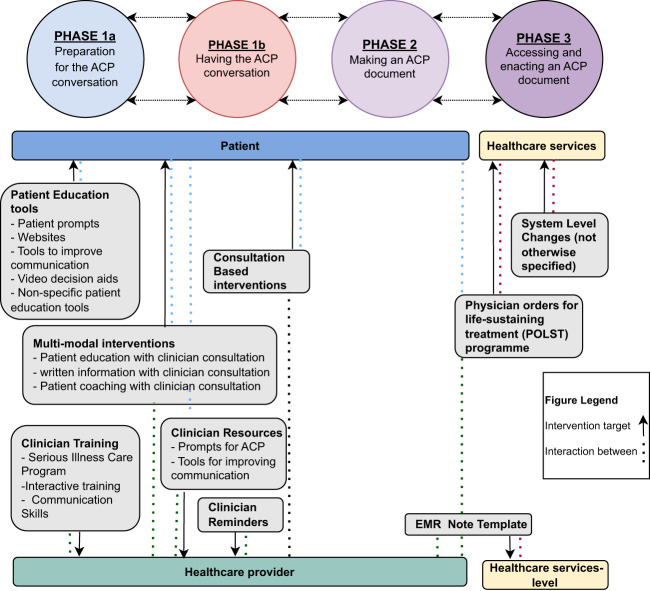
Mapping of ACP interventions to the Australian National Framework for ACP phases.

Nine of the 18 reviews identified interventions that aim to improve ACP uptake at various phases and target either the patient, healthcare provider, or healthcare service levels (12, 28, 29, 31, 32, 38–40, 43). In **Figure 3**, interventions have been mapped to the phases of ACP as presented by the Australian National Framework for advance care planning documents (45), with the addition of a preparatory phase, labelled Phase 1a. This figure also depicts the intervention target and interactions associated with delivering the intervention.

### Interventions targeting the patient

Seven reviews reported interventions that targeted the patient (12, 28, 29, 38–40, 43). Reporting of intervention effectiveness varied. Patient education tools were effective in increasing ACP documentation. Interventions that involved websites, patient prompts and/or patient tools to improve communication resulted in increased discussions of end-of-life issues and patients asking more questions (12, 29, 39, 43). Video-decision aid interventions increased knowledge scores and patients were less likely to opt for life-sustaining care (12, 28, 38–40). Consultation-based interventions did not report any effectiveness in improving ACP (12).

Multimodal interventions did not result in changes to ACP documentation, healthcare utilization, patient quality-of-life, consultation length, or communication self-efficacy. However, patients’ willingness to discuss end-of-life care, patient-physician communication, and patient knowledge and confidence in decision-making were enhanced (29, 38, 43).

### Interventions targeting the healthcare provider

Three reviews reported interventions that targeted the healthcare provider (31, 32, 39). Interventions that used clinician resources reported an increase in ACP discussion (32, 39). Clinician reminders (email reminders to address goals of care) increased ACP documentation from 14.5% to 33.7% (39).

Interventions providing clinician training administered the Serious Illness care program (39), interactive training (31), or training to improve clinician communication (32). These interventions were associated with an increase in discussions, earlier initiation of ACP discussions, and an increase in clinician confidence in initiating ACP conversations. However, they had little impact on ACP documentation.

### Interventions targeting healthcare services

Three interventions targeted healthcare services (32, 39). Intervention effectiveness was not reported; however, an Advance Directive was documented for 33 of 48 patients, with the availability of an EMR note template (39).

## Synthesis with theoretical domains framework domains

**Table 5** compares the frequencies of Theoretical Domains Framework domains for barriers of ACP with the Theoretical Domains Framework domains for implied barriers targeted by ACP interventions across patient, healthcare provider, and healthcare systems levels. Across levels, there was a misalignment between barriers identified and implied barriers targeted by interventions. Interventions most frequently targeted the patient; however, more barriers for ACP were identified for healthcare providers. There were also implied barriers targeted by ACP interventions that were not identified as barriers to ACP in the included reviews. This occurred for interventions targeting the patient as well as the healthcare provider.

**Table 5:** Frequencies of the Theoretical Domains Framework domains for barriers of ACP and barriers targeted by ACP interventions.

Frequency of domains coded	Theoretical Domains Framework domains for barriers to ACP	Alignment of barrier domains with targeted barrier domains for interventions	Theoretical Domains Framework domains for implied barriers targeted in ACP Interventions	Frequency of domains coded
9 8 7 6 5 2 1 1 1	**Patient** *Knowledge *Emotion *Social/professional roles and identity *Social influences *Beliefs about consequences *Environmental context and resources Memory attention and decision processes *Skills Goals	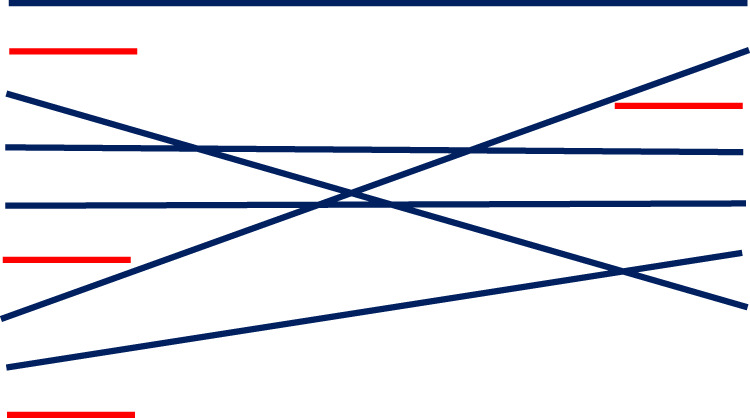	**Patient** *Knowledge Memory attention and decision processes Beliefs about capabilities *Social influences *Beliefs about consequences *Skills *Social/professional roles and identity	12 6 5 4 3 3 1
14 8 7 7 6 5 3 3 3 2 1 1	**Healthcare provider** *Beliefs about consequences *Knowledge *Emotion *Skills *Social/professional roles and social *Environmental context and resources Behavioural regulation *Social influences Memory attention and decision processes Intentions Optimism Goals	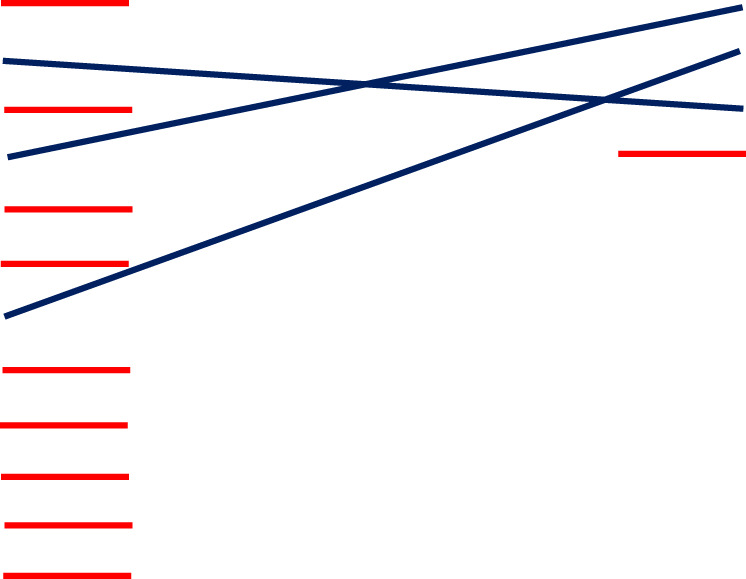	**Healthcare provider** *Skills Behavioural regulation *Knowledge Beliefs about capabilities	3 2 1 1
3	**Healthcare services** *Environmental context and resources	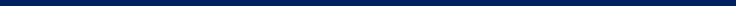	**Healthcare services** *Environmental context and resources	3

*Denotes domains of importance; 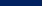 represents domains for barriers aligned with implied barriers targeted in ACP interventions; 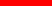 represents domains for barriers that are not aligned with a corresponding domain for a barrier/implied barrier targeted in an ACP intervention.

### Summary of results

- Five common categories were identified when defining ACP. However, these were not consistently applied across reviews, and there was no emergence of a clear definition of ACP over time.
- The most common combination of categories/subcategories used in defining ACP were: the purpose of ACP is to make decisions; patients should have conversations with family and healthcare providers; conversation should cover care options; and ACP should result in documentation (in the form of a legal document).
- There were more proposed than empirically supported benefits for ACP. There were no proposed or empirically supported benefits for the healthcare provider.
- A greater number of barriers for ACP were associated with the healthcare provider than the patient, or healthcare service. Enablers of ACP were greater for the patient than the healthcare provider or service.
- The majority of interventions to improve ACP target the patient rather than healthcare providers. Implied barriers that were targeted by ACP interventions and coded to Theoretical Domains Framework domains did not align with barriers identified in the included reviews as the most important in influencing ACP.
- Theoretical Domains Framework Effectiveness of ACP interventions varied. Interventions targeting identified barriers tended to be more effective.

## Discussion

**Figure 4:**
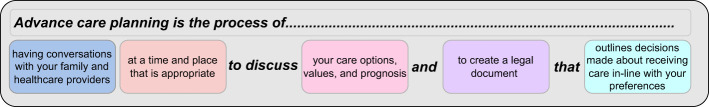
Key categories for defining ACP in oncology settings.

Based on this systematic overview of reviews, consistency is lacking in the literature in relation to defining ACP, its benefits, and its barriers and enablers in oncology settings. While the peer-reviewed literature lacks a consensus definition, there are key categories and sub-categories that align with the benefits of ACP and overarching values associated with optimal patient care that should be consistently used in its definition. The most frequently used sub-categories to define the purpose of ACP are about making decisions to ensure that the patient receives care in-line with their preferences. Receiving care that is in-line with one’s preferences and values is the hallmark of patient-centered care (46) and known to improve care quality and patient satisfaction (47). It is also one of the empirically supported benefits of ACP (28, 41). We suggest that these content categories should be included in the standardized definition of ACP (presented in **Figure 4**), along with identifying who should participate in the conversation. Evidence suggests the involvement of family and healthcare providers in ACP conversations is an enabler for the patient and healthcare provider for ACP uptake (27, 41–43).

The lack of consensus around the timing of ACP should be addressed within oncology settings, as this is also associated with barriers for healthcare providers not knowing when to initiate the conversation (32, 41, 43). It is important to consider that, within this patient population, the timing of conversations does not necessarily have a negative impact on patients (40) but, rather, consideration of contextual factors is important, such as having the conversation in an appropriate and private setting important (27, 29, 34). Whilst there is agreement within the literature (12, 28, 30, 31, 35, 37, 41–43) and also in the Australian National Framework of ACP (45), that ACP should result in a legal document, emphasizing the importance of this step, we found no mention of barriers associated with creating this document or any process or person to facilitate this process. Nor did any interventions target this phase of ACP.

Interventions predominantly focused on a preparatory phase of ACP, which we identified as Phase 1a (**Figure 3**): Preparation for the ACP conversation; currently beyond the scope of the Australian National Framework of ACP, which primarily focusses on three phases ACP; 1) having the conversation; 2) making an ACP document; and 3) accessing and enacting an ACP document (45). Interventions to enhance the uptake of ACP sometimes, but not always, addressed the known barriers and there appeared to be considerable variation in these interventions to improve ACP uptake. They also tended to target the patient rather than healthcare providers, even though the number of barriers associated with healthcare providers were a third greater than those for patients.

Further expanding the ‘importance criteria’ to a theme level enabled us to identify the mismatch of interventions in targeting empirically identified problems. Interventions that targeted patients did address patient barriers that were coded to important domains, and to some extent were effective in increasing ACP documentation. However, these interventions aimed to improve the knowledge of patients on end-of-life care decisions and gaining medical knowledge, yet the most important knowledge enabler was for patients to have an understanding of their prognosis. Interventions also focused on communication between patients and clinicians. While these interactions are important, the involvement of family members in the process of ACP was an enabler for both patients and healthcare providers. Yet, no interventions focused on educating and actively engaging family members in ACP. This is despite empirically supported psychological benefits and satisfaction with care being linked to the involvement of family members (28, 41).

Few interventions targeted empirically identified problems for healthcare providers, and these were mostly ineffective in increasing ACP documentation. These interventions targeted barriers coded to only two of the seven domains identified as important in influencing ACP uptake for healthcare providers (i.e., skills and knowledge); and were placed in phase 1 of the Australian National Framework of ACP (45). Interventions have failed to address the most frequently reported barriers for healthcare providers, specifically, beliefs that ACP conversations would have a negative impact on patients. This is in spite of patient accounts that this assumption is incorrect and contrary to empirically identified benefits for patients (40).

The pathways from having the ACP conversation to phase 2 of the Australian National Framework of ACP, making an ACP document, were not discussed in the literature reviewed in this overview. The barriers and enablers of making an ACP document have not been explored in the literature, nor addressed in any interventions. Yet, national frameworks identify this as a phase of successful ACP, consistent with many definitions that state ACP should result in some form of documentation. Interventions addressing phase 3 of the framework, accessing and enacting an ACP document, did not report effectiveness in improving ACP. System-level constraints was one of three themes coded to the domain of environmental context and resources and identified as important in influencing ACP uptake.

In summary of the findings discussed above, we recommend that future ACP interventions and research focus on:

- Interventions that target educating family members and actively engaging family in ACP.
- Interventions that encourage the discussion and understanding of prognosis;
- Interventions that challenge clinician beliefs— about understanding the impact and benefits of ACP; and
- The importance of context and availability of resources.

### Limitations of this research

While there is the lack of emergence of a clear definition of ACP in the academic literature, governments and non-governmental organizatisations may employ more complete definitions that were not included in this review; such as the one proposed in the Australian National Framework of ACP (45). The scope of the inclusion criteria for this overview may have also excluded other interventions for ACP that were not trialed only in cancer populations and, therefore, were not included in this analysis. It is possible that additional barriers and enablers of ACP, as well as potentially effective interventions, may also be relevant for cancer populations but have not been identified or included in this review.

In conclusion, this overview of reviews has identified key categories of content that should be included in defining ACP. These address the most frequently used sub-categories and are consistent with empirically supported benefits of ACP. We have also identified that, in many cases, proposed benefits of ACP did not actualize into empirically supported benefits. This was most evident for empirically supported benefits for patients and family members. No benefits of ACP were reported in the literature for healthcare providers. Lastly, interventions tended to target a different population and barriers than the ones the majority of evidence identified as a problem. Implications for this are that, in targeting an imagined problem as opposed to one that has been empirically identified we are unlikely to be effective in changing ACP uptake. Future interventions for ACP should target the domains of importance identified and address key barriers to change the behaviours of healthcare providers and improve ACP uptake.

## Data availability statement

The original contributions presented in the study are included in the article/**Supplementary Material**. Further inquiries can be directed to the corresponding author.

## Author contributions

LG conducted the search. Authors LG and SF screened reviews for inclusion and conducted the data extraction. LG, JF, AH and KG participated in the analysis of results. Writing of the original draft manuscript was prepared by LG. All authors contributed to the article and approved the submitted version.
