# Supplementary material for: Why is advance care planning underused in oncology settings? A systematic overview of reviews to identify the benefits, barriers, enablers, and interventions to improve uptake
Source: Front Oncol. 2023 Apr 28;13:1040589. doi: 10.3389/fonc.2023.1040589 (PMC10175822; doi:10.3389/fonc.2023.1040589)
Supplement: Supplementary file 1 [file Table_1.docx]

Supplementary File 1. Australian advance care planning terms of reference by state

| **State/Territory** | **Substitute decision maker** | **Advance Care Directive** |
| --- | --- | --- |
| ACT | Enduring Power of Attorney | Health Direction and Statement of Choices |
| NSW | Enduring Guardian | Advance Care Directive |
| NT | Decision Maker | Advance Personal Plan |
| Queensland | Enduring Power of Attorney | Advance Health Directive and Statement of Choices |
| South Australia | Substitute Decision Maker | Advance Care Directive |
| Tasmania | Enduring Guardian (must be registered) | Advance Care Directive |
| Victoria | Medical Treatment Decision Maker | Advance Care Directive |
| Western Australia | Enduring Power of Guardianship | Advance Health Directive and My Advance Care Plan |

International variations of advance care planning terms of reference

|  | **Substitute/surrogate decision maker** | **Advance care directive** |
| --- | --- | --- |
| USA examples | Durable Power of Attorney for Health Care (DPAHC)  Health Care Proxy Designations  Health Care Power of Attorney  Medical Power of Attorney | Living Will  Advance Directive |
| UK | Lasting Power of Attorney for Health and Welfare  Power of Attorney | Advance Decision to refuse treatment  Living Will (before 2007)  Advance Statement  Advance Directive |
| New Zealand | Enduring Power of Attorney | Advance Care Plan |
| Europe | Various including personal representative, surrogate decision maker | Various, including advance care directive |
| Canada | Substitute decision maker | Advance care plan |
