# Supplementary material for: Why is advance care planning underused in oncology settings? A systematic overview of reviews to identify the benefits, barriers, enablers, and interventions to improve uptake
Source: Front Oncol. 2023 Apr 28;13:1040589. doi: 10.3389/fonc.2023.1040589 (PMC10175822; doi:10.3389/fonc.2023.1040589)
Supplement: Supplementary file 2 [file DataSheet_1.docx]

**Supplementary File 2: Search Terms**

1. “Advance care plan*”
2. “advance directive*”
3. “living will”
4. 1 or 2 or 3
5. Neoplasm*
6. Cancer
7. Carcino*
8. Leukaemia* or Leukemi*
9. Tumour* or tumor*
10. Malignan*
11. Lymphoma*
12. Adenocarcinoma*
13. Metastat*
14. Sarcoma*
15. Oncolog*
16. 5 or 6 or 7 or 8 or 9 or 10 or 11 or 12 or 13 or 14 or 15
17. 4 and 16
18. Review (title)
19. Review (publication type)
20. Overview (title)
21. 18 or 19 or 20
22. 21 AND 17
