# Supplementary material for: Why is advance care planning underused in oncology settings? A systematic overview of reviews to identify the benefits, barriers, enablers, and interventions to improve uptake
Source: Front Oncol. 2023 Apr 28;13:1040589. doi: 10.3389/fonc.2023.1040589 (PMC10175822; doi:10.3389/fonc.2023.1040589)
Supplement: Supplementary file 3 [file DataSheet_2.doc]

**Supplementary File 3**

**Thematic synthesis of the Theoretical Domains Framework** **domains identified as barriers and enablers of ACP**

***Knowledge (10 reviews of the 15 reviews that addressed barriers/enablers of ACP, 5/7 reviews with expressed importance)***

*Theme with expressed importance: Understanding of ACP and its use or non-use (6 reviews):*

Understanding of the purpose of ACP was the most frequently coded theme in this domain (29%) and also identified with expressed importance. A barrier to successful ACP for patients is a lack of understanding of the purpose of ACP: “*ADs may* *fail to achieve their goals when the patient and family members do not understand how they will be used or their goal”* [27]. Healthcare providers identified this as an important barrier to address in order to have patients actively engage in ACP, *“highlighted the importance of actively educating patients prior to the regular oncology consultation to enhance their ‘motivation’ and ‘competence’ to take part in an ACP discussion rather than just providing information on ACP to them”* [24]. A lack of understanding of ACP also impacted healthcare provider engagement *“Health professionals’ knowledge of … ACP was also consistently found to be an important factor in their willingness to initiate or participate in ACP”* *[22].*

*Theme with expressed importance: Patient understanding, or lack of understanding, of their prognosis (5 reviews):* The patient’s understanding of their prognosis, or lack of understanding, was an enabler and a barrier, respectively, for both the patient and the healthcare provider [24,27,29,33,38]. This theme accounted for 21% of the coded barriers and enablers for this domain. Healthcare providers reported this influencing their engagement in ACP “*physicians are more likely to discuss end of life ( EOL)‐care preferences with cancer patients who accurately understood their prognosis”* [29]. Realistic expectations about prognosis were identified as important in influencing patient engagement in ACP: “*main reason patients fail to complete their directives is the difficulty in anticipating their wills based on scenario projections”* [27].

A *procedural understanding* of when, how, and what to discuss as part of ACP, was both a barrier and enabler for healthcare providers [18, 23, 27]. Relative *certainty of the prognosis* of a patient influences engagement of healthcare providers in ACP and acts as a barrier for ACP [23] and an enabler: *“accurate estimates of life expectancy to within a year is associated with greater illness awareness and higher rates of ACP”* [28].

*Patient understanding of their disease* was a barrier for the patient but not the healthcare provider [17, 27]. A *patients’ medical knowledge* however, was relevant to patients and healthcare providers with several studies reporting that this was an enabler for the patient and a barrier for both patients and healthcare providers: *“factors that impact the inefficiency of these ACP discussions…are often patients’ lack of sufficient medical knowledge to engage in these discussions”* [12].

*Clinician ignorance* of ACP is also a barrier: *“If physicians are ignorant of ACP … then, they are unlikely to encourage patients to pursue it”* [11].

***Skills (9 reviews, 4 with expressed importance)***

*Theme with expressed importance: Training on ACP or lack thereof (5 reviews):* The main barrier associated with the skills domain was a lack of formal training on ACP for healthcare providers [23,30,33,37,38] *“oncologists may not be prepared to help patients navigate these options and engage in ACP due to lack of formal training lack of formal training” [24].* Within this domain 46% of barriers and enablers were coded to this theme. Healthcare providers report feeling inappropriately trained or prepared to have ACP conversations. Trained facilitators were expressed as *“essential elements of ACP”* [24]

*Theme with expressed importance: Clinician communication skills (4 reviews)*: Communication skills accounted for 38% of barriers and enablers for patients and healthcare providers in this domain. Healthcare providers ability to facilitate discussions and develop rapport with patients were described important for opening up discussions about ACP [22,37].

*Palliative care skills* to aiding the discussions towards patient preparation and readiness for ACP was also reported as an enabler: *“palliative care can enhance prognostic discussions and ACP through a dedicated focus on eliciting patients’ values, illness understanding, and expectations for the future”* [29].

***Environmental context and resources (10 reviews, 4 reviews with expressed importance)***

*Theme with expressed importance: System level constraints (5 reviews):* System level constraints in accessing ACP documentation from a healthcare services perspective was coded as a barrier and enabler for ACP (37% of coded themes in this domain) [24,27,28,30,37]. System constraints and availability of resources were reported as important factors influencing ACP *“most importantly a supportive contextual environment (e.g. availability of administrative system, sufficient resources… should be in place to support the implementation”* [24].

*Theme with expressed importance: Time constraints (4 reviews):* Time constraints associated with outpatient and inpatient settings was reported as a barrier for patients and healthcare providers accounting for 37% of coded themes in this domain [22,33,36,38]. Healthcare providers report this barrier limits the scope of ACP conversations and the “*input of a physician or nurse who understands and is aware of the cognitive or emotional challenges pertaining to each patient”* [38]. Patients expressed importance that *“ACP conversations should be initiated with adequate time and place for reflection”* *[27]* emphasizes the influence of this theme.

*Theme with expressed importance: Appropriateness of setting (3 reviews):* The appropriateness of settings and if these settings provided adequate privacy was reported as both a barrier and enabler for healthcare providers, as well as for patients [22,24,29] (26%). The importance of privacy is emphasized by healthcare providers; *“physicians reported time and privacy as barriers to ACP, they did so because they believed these were fundamental to establishing relationships with patients and families”* [22].

***Social Influences (8 reviews, 4 with expressed importance)***

*Theme with expressed importance: Clinician engagement in ACP conversation (3 reviews)*: Involvement of healthcare providers in ACP conversations was the most frequently coded enabler of ACP for patients and of expressed importance. Patients preference for engaging in ACP conversations were with *“physician who knows them best [20], preferred that their physicians initiated discussion regarding ACP, and were more likely to participate in ACP or draw up an AD if they had discussed this with their oncologist”* [22].

*Theme with expressed importance: Family participation in ACP conversations (2 reviews)*: Family participation in ACP conversations was as an important enabler of ACP for healthcare providers and patients. Engaging family members in ACP conversation were reported *“essential elements of ACP for success”* [37]. Family participation was also found to be a barrier of ACP for patients, *“for some, including family in EOL decision-making raised concerns over upsetting or placing undue stress or burden on family members”* [22].

*Theme with expressed importance: Exclusion from ACP conversations (3 reviews)*: Family members excluding patients from ACP conversations is a barrier of ACP for patients and one of expressed importance *“it is very important to reinforce that the directives should be ideally created by the patient themselves”* [27]. Discussions of EoL care that exclude the healthcare provider were also identified as barriers for patients [33].

*Theme with expressed importance: Institutional culture (2 reviews):* The culture of an institution can also influence ACP and was identified as a barrier for both the patient and healthcare provider. This theme was considered important and reported to *“strongly influence….the behaviour and choices of patients, their loved ones, and the staff caring for them in relation to EOL”* [22].

The influences from *interactions with other colleagues* that share responsibility of the patient were reported to be enablers of ACP for healthcare providers [20].

***Beliefs about consequences (7 reviews, 2 with expressed importance)***

*Theme with expressed importance: Discussion would have a negative impact (6 reviews):* Healthcare provider concerns that initiating ACP conversations would have a negative impact on the patient accounted for 30% of the coded barriers within this domain. Healthcare providers reported this as the most common barrier [22,27,35-38]; *“ACP is sometimes stressful for patients”* [37]. Expressed importance was associated with this theme in the context of the patient voice which contradicts this belief; patients desire to be informed and *“important to bear in mind that the majority of patients do not complain about additional depression/anxiety after discussions about prognosis”* [27].

*Theme with expressed importance: Past experiences and attitudes towards the healthcare system (2 reviews):* Past experiences and attitudes towards the healthcare system was coded as a barrier and enabler of ACP for both patients and healthcare providers [22,36]. This was the most commonly coded theme within this domain (26%). Patients reported *“beliefs … towards healthcare, and this concept can affect the willingness of a person with full capacity in taking part in an ACP”* and *“in general… prior experience of EOL care or decision making are considered facilitators”* [36]; as influencing their engagement with ACP. Similarly the case for healthcare providers where previous experiences and attitudes *“ influence their support for and willingness to engage in ACP”* and was expressed as an *“important factor in their willingness to initiate or participate in ACP”* [22].

They also held a perception that initiating ACP conversations would *damage the patient physician relationship*: *“Further, some health professionals expressed concerns that ACP may damage their relationships with patients and were concerned that formal ACP ‘did not take account of professional–patient relationships and individual patient needs”* [20]; and that patients/families were *not accepting of their prognosis* and rather preferred to *maintain hope*: *“reasons nurses did not discuss prognosis and hospice referral with their patients were unwillingness of patients or families to accept prognosis .. and nurses’ desire to maintain hope for patients and their families”* [28]. Furthermore, nurses report *beliefs that patients and families do not want to engage in a conversation* of EoL with them [28], and that there will be repercussions from doctors for initiating ACP conversation with patients [20, 28].

Beliefs held by patients that were barriers for ACP were the uncertainty that ACP would be useful, or the perception ACP would impact the quality of care they received: *“denial that ACP would be useful, and perceptions that signing an AD would result in inferior care and that patients would be treated differently if they had an AD”* [28]. Patients also reported the perception that ACP conversations would be upsetting for their family and therefore acted as a barrier [20]. Beliefs about patient readiness and having conversations at the wrong time was the single theme within this domain that was reported as both a patient enable [34] and a healthcare provider barrier [25].

***Professional role and social identity (8 reviews, 2 with expressed importance)***

*Theme with expressed importance: Role clarity or a lack of (3 reviews):* Clarity on roles and who is responsible for initiating ACP was reported as both a barrier for healthcare providers [22,28,34]. Healthcare provider described the *“difficulty of implementing an ACP intervention on a larger scale is determining which member of the health care team will be responsible for ACP”* [34]. Patient expressed preference emphasizes the importance of this theme *“patients generally preferred to do ACP with the physician who knows them best, preferred that their physicians initiated discussion regarding ACP, and were more likely to participate in ACP”* [22].

Nurses in particular reported a perceived *lack of authority* as a barrier in initiating ACP [20], as well as a *perception that others, such as patients, patient families and doctors, did not think it was the nurses role* to initiate ACP discussions: *“the most commonly reported barriers to engaging in ACP with their patients were from patients’, their families’, and physicians’ reluctance to have these discussions with them”* [28]. Yet the most reported enabler for healthcare providers was that ACP required a *multidisciplinary approach* [8, 28, 36]. *Cultural and religious beliefs* were the most reported patient barriers to ACP: *“high levels of religiosity were associated with a lower uptake of ACP and a higher likelihood of aggressive treatment and death in ICU”* [27]; *“ACP is culturally sensitive and that the process and content of ACP could be different for countries that differ with respect to legal, cultural and religious issues”* [30].

***Emotion (10 reviews, 1 with expressed importance)***

*Themes with expressed importance: Acceptance of prognosis; and diminishing hope (9 reviews):* An unwillingness of patients or their families to accept their prognosis and a fear of diminishing hope are barriers for healthcare providers that were expressed as the *“top reasons”* that prevented them from engaging in ACP [30];*“While patients prefer honesty from their health care providers, how best to communicate with patients about these issues without compromising their sense of hope and optimism remains a significant challenge for those providers”* [35]

Several reviews reported the impact of emotion as a barrier for healthcare providers. *Fear of impacting the therapeutic relationship*: *“fearful of losing their therapeutic alliance”* [31]; as well as *fear of addressing bad news*  [25], were all barriers of ACP for healthcare providers. *Patient stress and anxiety* was reported as a barrier for both healthcare providers and patients [20, 25, 27]. *Patient fear, and fear for their family members* were also additional barriers for patients [20]. It was also reported that patients acknowledge a barrier of ACP to be their *perceived physician discomfort* in initiating ACP discussions: *“patients and caregivers explicitly acknowledged physician discomfort in discussing and initiating EOL conversations as a barrier to participating in discussions”* [20].

***Memory, attention and decision processes (4 reviews)***

Several reviews identified that decisions *not to disclose a poor prognosis* to the patient, were a barrier of ACP for healthcare providers [25, 27, 34]. Healthcare providers also reported having to decide on *when is the most appropriate time* to initiate ACP discussions was also challenging [25, 26]: *“determining the best timing to initiate ACP with different cancer stages and prognoses is challenging”* [26]. Ultimately for patients, their *capability to make decisions* was both a barrier and enabler to ACP: *“another problem in composing a directive may be the lack of time due to the rapid evolution of the disease accompanied by cognitive impairment and worsening of the general health condition”* [25].

***Optimism (1 review)***

The *desire to remain optimistic* was reported as a barrier [28]. Healthcare providers will report not wanting to discuss end of life unless a patient is near death: “*Providers may be overly optimistic, avoid these discussions, communicate with euphemisms, and delay conversations until patients are near death”.*

***Goals (1 review)***

Healthcare providers and patients reported *waiting for ACP to become relevant* before initiating ACP conversations: *“stakeholders are reluctant to initiate ACP early and prefer to delay ACP until the issues raised, particularly those surrounding preferences for EOL care, are more clinically relevant and appear more ‘real’, or more salient”* [20].

***Emotion (9 reviews)***

***Behavioural regulation (4 reviews)***

Healthcare providers reported that having no established guidelines for the timing of ACP discussions was a barrier for ACP : [26, 31, 32]: “conversations are not taking place until the last few months of life and often occur in the inpatient setting; however, this new practice expectation has not been accompanied by recommendations of exactly how ACP should be implemented in individual practices” [32]; *“there is no established paradigm to guide the timing, approach, and content of such discussions, which therefore often occur late if at all”* [31].

***Intentions (2 reviews)***

Reluctance to initiate ACP conversations with the patient early has been reported as a barrier of ACP for healthcare providers [26, 33]: *“providers were reluctant towards early initiation of ACP”* [26].
